# IDH, Recursos Humanos e Tecnológicos para o Diagnóstico e Tratamento das Malformações do Aparelho Circulatório no Brasil - Análise da Realidade no Brasil

**DOI:** 10.36660/abc.20210496

**Published:** 2021-07-15

**Authors:** Isabel Cristina Britto Guimarães

**Affiliations:** 1 Universidade Federal da BahiaFaculdade de Medicina da BahiaSalvadorBABrasilUniversidade Federal da Bahia - Faculdade de Medicina da Bahia, Salvador, BA - Brasil

**Keywords:** Doença Cardiovascular, Cardiopatias Congênitas, Serviço de Saúde Pública, Mortalidade Infantil, Mortalidade Perinatal

No Brasil, em 2019, as doenças do aparelho circulatório (DAC) foram a principal causa de morte.^1^ Em crianças menores de um ano, a DAC é a nona causa de morte e, na faixa etária de 1 a 9 anos, a oitava.^2^ Nas últimas décadas, houve uma redução da taxa de mortalidade infantil para 11,9 / 1.000 nascidos vivos (NV) em 2019.^3^ Apesar da redução progressiva observada nos últimos anos, essa taxa continua elevada quando comparada à de países desenvolvidos. Na Suécia, a taxa de mortalidade infantil no período de 2015/2020 foi de 2,0/1.000 NV. No Brasil, em crianças menores de cinco anos, a mortalidade foi de 14,0/1.000 NV, ocorrendo principalmente no primeiro ano de vida.^3^

As principais causas de mortalidade neonatal são asfixia perinatal, prematuridade extrema e malformações congênitas. Dentre as malformações congênitas (MC), as DAC têm maior impacto na mortalidade, por serem classificadas como causas de morte evitáveis, que poderiam ser reduzidas através de intervenções precoces.^4^ As altas taxas de mortalidade por malformações do aparelho circulatório (MAC) decorrem da falta de diagnóstico pré e pós-natal, resultando em tratamento inadequado e maior número de óbitos.^4-8^

O estudo de Salim et al. objetivou verificar a associação do diagnóstico de MAC ao nascimento e óbito por MAC no primeiro ano de vida com o índice de desenvolvimento humano (IDH) e recursos tecnológicos e humanos para o diagnóstico e tratamento de MAC por macrorregiões do Brasil, utilizando dados obtidos junto ao DATASUS, no período de 2000/2015.^9^

Entre 2000/2015, os autores encontraram uma taxa de MC de 660,8/100.000 NV, sendo 18.444 devido a MAC, e uma taxa de diagnóstico de 38,55/100.000 NV. Considerando as cinco macrorregiões brasileiras, as regiões Sul e Sudeste apresentaram as maiores taxas de diagnóstico de MAC ao nascimento e óbito por MAC, enquanto as regiões Norte e Nordeste apresentaram as menores taxas de diagnóstico de MAC ao nascimento e óbito por MAC no primeiro ano de vida. A região Centro-Oeste, com recursos semelhantes aos das regiões Sul e Sudeste, apresentou baixo índice de diagnóstico de MAC ao nascimento e melhora no diagnóstico de MAC após o óbito. Esses dados são preocupantes, demonstrando o impacto negativo que as desigualdades sociais têm na atenção à saúde da população, onde os melhores resultados são descritos nas regiões com maiores valores de IDH, recursos tecnológicos e humanos. Entretanto, as taxas nacionais de diagnóstico de MC e MAC são ainda menores quando comparadas às taxas globais no mesmo período e para a mesma faixa etária: 1,40 e 1,78, respectivamente.^3^ As diferenças permanecem quando comparadas aos países desenvolvidos, onde há maior investimento em recursos de saúde.^3,10^

Ainda vivenciamos uma lacuna significativa no número de cirurgias cardíacas pediátricas realizadas anualmente no país.^11^ Considerando uma prevalência de cardiopatia congênita (CC) de 9: 1.000 NV, a estimativa é de 28.846 casos novos / ano de CC, dos quais aproximadamente 50% necessitarão de cirurgia cardíaca no primeiro ano de vida, correspondendo a uma necessidade média de cirurgia cardíaca em CC de cerca de 23.077 procedimentos/ano.^12^ Pinto Jr et al., ao analisarem a situação da cirurgia cardiovascular brasileira quanto ao número de procedimentos realizados em 2002, encontraram um déficit de 65%, sendo as maiores taxas sendo encontradas nas regiões Norte e Nordeste (93,5% e 77,4%, respectivamente) e os menores nas regiões Sul e Centro-Oeste (46,4% e 57,4%, respectivamente).^11^

Caneo et al. compararam a necessidade e o número de cirurgias cardíacas realizadas no primeiro ano de vida em 2010 entre o estado de São Paulo e a Polônia, que possuíam IDH e tamanho populacional semelhantes à época. Na Polônia, crianças menores de 1 ano foram submetidas à 100% das cirurgias necessárias, enquanto em São Paulo, apenas 50% o fizeram. Isso demonstra que mesmo em num dos principais centros de cirurgia cardíaca do país, ainda temos um longo caminho a percorrer em termos de diagnóstico e intervenção precoces.^13^

Vale destacar alguns aspectos do estudo de Salim et al., como maior mortalidade por MAC nas regiões Norte, Nordeste e Centro-Oeste, entre os casos de MC no primeiro ano de vida.^7,9^ Isso ocorre devido a diversas condições: a CC considerada crítica requer diagnóstico pré e pós-natal precoce, assistência ao parto e acesso garantido a centros de referência para os casos indicados, o que requer recursos humanos e tecnológicos especializados.^7,8,11,14^ De acordo com os dados encontrados, existe um significativo déficit de recursos humanos com formação adequada para realizar o diagnóstico e o atendimento de crianças com cardiopatia, principalmente nas regiões Norte, Nordeste e Centro-Oeste, relacionado não apenas aos pediatras, mas também aos cardiologistas pediátricos com formação em ecocardiografia, cirurgiões cardiovasculares e disponibilidade de centros cirúrgicos cardiológicos pediátricos por macrorregião.

A redução da mortalidade infantil ainda é um grande desafio no Brasil para os governos, profissionais de saúde e sociedade como um todo. É necessário o aprimoramento das estratégias de saúde pública e maior atenção ao recém-nascido, visando obter o diagnóstico precoce e tratamento das cardiopatias congênitas.^14^

De acordo com Ottersen et al., o objetivo da política de equidade em saúde não é eliminar todas as diferenças de saúde para que todos tenham a mesma qualidade de atendimento, mas reduzir ou eliminar aquelas decorrentes de fatores considerados evitáveis e injustos.^15^
