# Supplementary material for: IDH, Recursos Humanos e Tecnológicos para o Diagnóstico e Tratamento das Malformações do Aparelho Circulatório no Brasil - Análise da Realidade no Brasil
Source: Arq Bras Cardiol. 2021 Jul 15;117(1):72–3. [Article in Portuguese] doi: 10.36660/abc.20210496 (PMC8294724; doi:10.36660/abc.20210496)
Supplement: Translation

# Translation: HDI, Technological and Human Resources for the Diagnosis and Treatment of Circulatory System Malformations in Brazil - Analysis of the Reality in Brazil

**Authors:** Isabel Cristina Britto Guimarães

**Affiliations:** 1 BrasilUniversidade Federal da Bahia - Faculdade de Medicina da Bahia, Salvador, BA - Brasil

**Keywords:** Cardiovascular Disease, Heart Defects, Public Health Service, Infant Mortality, Perinatal Mortality

In Brazil in 2019, circulatory system diseases (CSD) were the leading cause of death.^1^ In children under one year of age, CSD is the ninth leading cause of death and, in the age group from 1 to 9 years old, the eighth.^2^ In recent decades, there has been a reduction in the infant mortality rate to 11.9/1,000 live births (LB) in 2019.^3^ Despite the progressive reduction observed in recent years, this rate remains high when compared to that in developed countries. In Sweden, the infant mortality rate in the period of 2015/2020 was 2.0/1,000 LB. In Brazil, in children under five years of age, mortality was 14.0/1,000 LB, which occurred mainly in the first year of life.^3^ The main causes of neonatal mortality are perinatal asphyxia, extreme prematurity, and congenital malformations. Among the congenital malformations (CM), CSD have the greatest impact on mortality, as they are classified as preventable causes of death, which could be reduced through early interventions.^4^ High mortality rates due to malformations of the circulatory system (MCS) result from the lack of pre and postnatal diagnosis, resulting in inadequate treatment and a higher number of deaths.^4-8^

The study by Salim et al. aimed to verify the association of MCS diagnosis at birth and death from MCS in the first year of life with the human development index (HDI) and technological and human resources for the diagnosis and treatment of MCS by the macroregions of Brazil, using data obtained from DATASUS, in the period of 2000/2015.^9^

Between 2000/2015, the authors found a CM rate of 660.8/100,000 LB, being 18,444 due to MCS, and a diagnosis rate of 38.55/100,000 LB. Considering the five Brazilian macro-regions, the South and Southeast regions had the highest rates of diagnosis of MCS at birth and death from MCS, while the North and Northeast regions had the lowest rates of diagnosis of MCS at birth and death from MCS in the first year of life. The Midwest region, with resources similar to those of the South and Southeast regions, had a low rate of diagnosis of MCS at birth and an improvement in the diagnosis of MCS after death. These data are a matter of concern, demonstrating the negative impact that social inequalities have on the population’s health care, where the best results are described in the regions with the highest HDI values, technological and human resources. However, the national rates of CM and MCS diagnosis are even lower when compared to the global rates in the same period and for the same age group: 1.40 and 1.78, respectively.^3^ Differences remain when compared to developed countries, where there is greater investment in health care resources.^3,10^

We still experience a significant gap in the number of pediatric cardiac surgeries performed annually in the country.^11^ Considering a prevalence of congenital heart disease (CHD) of 9:1,000 LB, the estimate is of 28,846 new cases/year of CHD, of which approximately 50% will need cardiac surgery in the first year of life, corresponding to an average need for cardiac surgery in CHD of around 23,077 procedures/year.^12^ Pinto Jr et al., when analyzing the situation of Brazilian cardiovascular surgery, regarding the number of procedures carried out in 2002, found a deficit of 65%, with the highest rates being found in the North and Northeast regions (93.5% and 77.4%, respectively) and the lowest ones in the South and Midwest regions (46.4% and 57.4%, respectively).^11^

Caneo et al. compared the need and number of cardiac surgeries performed in the first year of life in 2010 between the state of São Paulo and Poland, which had similar HDI and population size at the time. In Poland, children under 1 year of age underwent 100% of the necessary surgeries, while in São Paulo, only 50% did. This demonstrates that even in one of the main cardiac surgical centers of the country, we still have a long way to go in terms of early diagnosis and intervention.^13^

It is worth highlighting some aspects in the study by Salim et al, such as a higher mortality rate from MCS in the North, Northeast, and Midwest regions, among cases of CM in the first year of life.^7,9^ This occurs due to several conditions: the CHD considered critical requires early pre and postnatal diagnosis, childbirth care, and guaranteed access to reference centers for the indicated cases, which requires specialized human and technological resources.^7,8,11,14^ According to the data found, there is a significant deficit of human resources with adequate training to carry out the diagnosis and care of children with heart disease, especially in the North, Northeast, and Midwest regions, related not only to pediatricians but also to pediatric cardiologists with training in echocardiography, cardiovascular surgeons and the availability of pediatric cardiac surgical centers by macroregion.

The reduction of infant mortality is still a major challenge in Brazil for governments, health professionals, and society as a whole. The improvement of public health strategies and greater attention to newborns, aiming at early diagnosis and treatment of congenital heart disease is necessary.^14^

According to Ottersen et al., the objective of the policy for equity in health is not to eliminate all differences in health so that everyone has the same quality of care, but to reduce or eliminate the ones that result from factors considered preventable and unfair.^15^
